# *Cochylis* Treitschke in China: one new species and five new records (Lepidoptera, Tortricidae, Cochylini)

**DOI:** 10.3897/zookeys.258.4108

**Published:** 2013-01-15

**Authors:** Yinghui Sun, Houhun Li

**Affiliations:** 1 College of Life Sciences, Nankai University, Tianjin 300071, P. R. China

**Keywords:** Lepidoptera, Tortricidae, Cochylini, *Cochylis*, new species, new record, China

## Abstract

Six species of *Cochylis* Treitschke, 1829 are recorded for China. Among them, *Cochylis triangula* **sp. n.** is described as new; *Cochylis atricapitana* (Stephens, 1852), *Cochylis discerta* Razowski, 1970, *Cochylis dubitana* (Hübner, [1799]), *Cochylis faustana* (Kennel, 1919) and *Cochylis posterana hyrcana* (Toll, 1948) are recorded for the first time for China. The female of *Cochylis discerta* Razowski, 1970 is described for the first time. Adults and genitaliaare illustrated, a key is given for the identification of Chinese species based on male genitalia.

## Introduction

The genus *Cochylis* Treitschke, 1829 belongs to the tribe Cochylini of the subfamily Tortricinae. *Cochylis* was erected by Treitschke (1829) for the type species *Tortrix roseana* Haworth, 1811. Brown (2005) listed 68 species and five subspecies. Subsequently, Brown (2006) described a new species from Argentina; Razowski and Wojtusiak (2006) described a new species from Venezuela; Razowski and Becker (2007a, b) described a new species from Argentina and Cuba respectively; Metzler and Forbes (2012) described a new species from the USA. *Cochylis nana* (Haworth, 1811) and *Cochylis voxcana* (Kearfott, 1907) were transferred to the genus *Thyraylia* Walsingham, 1897 (Gilligan et al. 2012). Currently, *Cochylis* consists of 71 species and five subspecies, distributed in the Holarctic, Oriental and Neotropical regions; 26 of these occur in the Palaearctic and Oriental regions.

Liu and Li (2002) recorded six *Cochylis* species from China, of which *Cochylis nana* (Haworth, 1811) was subsequently transferred to the genus *Thyraylia*. In this paper, we describe one new species and record five additional species for the Chinese fauna.

## Material and methods

This study is based on the examination of specimens collected by light traps. Morphological terminology follows Razowski (1987). Genitalia were prepared and mounted according to the methods introduced by Li (2002). Photos of the adults were taken with a Nikon D300 digital camera plus macro lens, and illustrations of the genitalia were prepared by using an Olympus C-7070 digital camera attached to an Olympus BX51 microscope. The examined specimens, including the types of the new species, are deposited in the Insect Collection, College of Life Sciences, Nankai University, Tianjin, China (NKUM). Type locality is abbreviated as TL.

## Taxonomy

### Cochylis

Treitschke, 1829

http://species-id.net/wiki/Cochylis

Cochylis Treitschke, 1829: 233. Type species: *Tortrix roseana* Haworth, 1811.
Chochylis Duponchel, 1836: 409. [misspelling of *Cochylis*]
Conchylis Sodoffsky, 1837: 93. [unjustified emendation of *Cochylis*]
Pontoturania Obraztsov, 1943: 97. Type species: *Cochylis defessana* Mann, 1861.
Acornutia Obraztsov, 1944: 68. Type species: *Tortrix nana* Haworth, 1811.
Cochylichroa Obraztsov & Swatschek, 1958: 233. Type species: *Eupoecilia atricapitana* Stephens, 1852.
Brevicornutia Razowski, 1960: 317. Type species: *Cochylis pallidana* Zeller, 1847.
Longicornutia Razowski, 1960: 314. Type species: *Tortrix (Cochylis) phaleratana* Herrich-Schäffer, 1851 sensu Razowski, 1960 [=*Cochylis epilinana* Duponchel, 1842]
Neocochylis Razowski, 1960: 316. Type species: *Conchylis calavrytana* Rebel, 1906.
Paracochylis Razowski, 1960: 316. Type species: *Cochylis amoenana* Kennel, 1899.

#### Diagnostic characters.

*Cochylis* is characterized by the combination of the following characters: adult small to medium; forewing with all veins separate, Sc reaching middle of the costal margin, basal distance between R_1_−R_2_ about three times of between R_2_−R_3_, R_5_ to the costal margin; hindwing with costal fold in male, Rs and M_1_ arising from the same point or long stalked, M_3_ and CuA_1_ separate, female hindwing usually with two spines in the frenulum; male genitalia: tegumen short and broad; uncus and gnathos absent; socius separate, drooped, connected with distal part of tegumen at base; median process of the transtilla mostly developed and dentate distally (absent in a few species); sacculus developed with a hook-shaped process basally in some species, with or without terminal process; slender vinculum separate ventrally; phallus without cornutus or with cornutus composed of a bundle of spines; female genitalia: sterigma varied in size and sclerotized diversely; antrum developed and heavily sclerotized; ductus bursae short, indistinctly distinguished from the corpus bursae; corpus bursae membranous, densely suffused with tiny spines.

#### Biology.

In the Palaearctic Region, there are one or two generations annually, and over-wintering occurs in the larval stage. Larvae are oligophagous and feed mainly on plants belonging to Asteraceae (Razowski 1987).

#### Distribution.

All species are distributed in the Holarctic, Oriental and Neotropical regions.

#### Key to Chinese species of *Cochylis* based on male genitalia

**Table:** 

1	Phallus uniformly slender	2
–	Phallus stout basally, slender distally	6
2	Phallus with cornutus (Razowski 1970: Taf. 106, Fig. 270)	*Cochylis atricapitana*
–	Phallus without cornutus	3
3	Transtilla without median process (Fig. 7)	*Cochylis discerta*
–	Transtilla with median process	4
4	Phallus slightly sinuate (Razowski 1970: Taf. 104, Fig. 260)	*Cochylis roseana*
–	Phallus curved orthogonally distally	5
5	Sacculus extended to a hook-shaped process basally (Razowski 1970: Taf. 105, Fig. 268_1–4_)	*Cochylis hybridella*
–	Sacculus not extended basally (Fig. 8)	*Cochylis dubitana*
6	Transtilla without median process (Razowski 1970: Taf. 109, Fig. 283)	*Cochylis psychrasema*
–	Transtilla with median process	7
7	Valva with a spine-shaped process at basal 1/3 near outer margin (Fig. 11)	*Cochylis triangula* sp. n.
–	Valva without process near out margin	8
8	Median process nearly triangular (Fig. 10)	*Cochylis posterana hyrcana*
–	Median process short stripe-shaped, nearly parallel-sided	9
9	Socius about 1/2 length of median process (Razowski 1970: Taf. 108, Fig. 279)	*Cochylis defessana*
–	Socius almost same length as median process	11
11	Valva with outer margin slightly protruded distally, costa straight (Fig. 9)	*Cochylis faustana*
–	Valva with outer margin nearly straight distally, costa slightly concave (Razowski 1970: Taf. 108, Fig. 277_1–2_)	*Cochylis piana*

### Cochylis atricapitana

(Stephens, 1852)

http://species-id.net/wiki/Cochylis_atricapitana

Figs 1 , 12

Eupoecilia atricapitana Stephens, 1852: 80. TL: England.
Cochylis atricapitana (Stephens, 1852): Razowski, 1960: 317.

#### Material examined.

**CHINA: Xinjiang Uyghur Autonomous Region:** 6 ♀♀, Buerjin (47°41'N, 86°59'E), 21.vii.2007, leg. Xinpu Wang.

#### Diagnosis.

**Figures 1–6.**
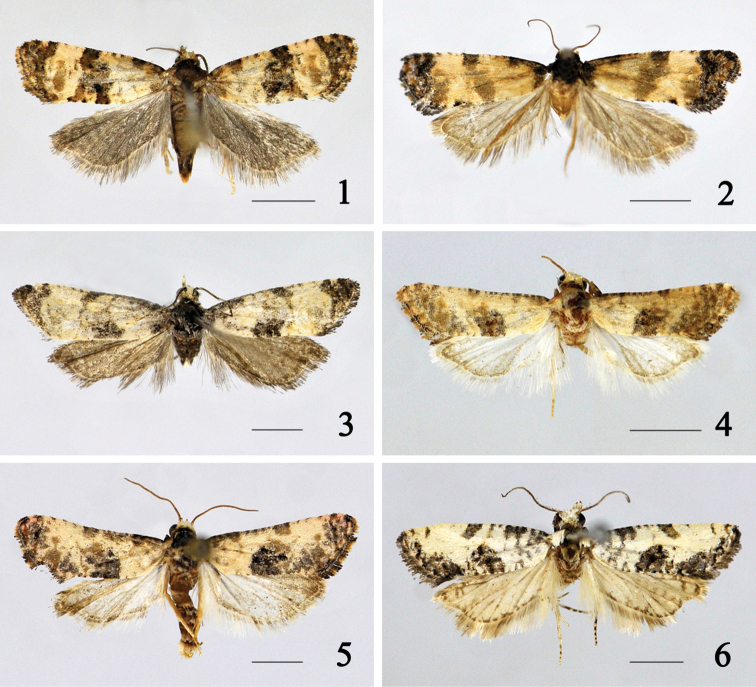
Adults of *Cochylis* spp. **1** *Cochylis atricapitana* (Stephens), female (Buerjin, Xinjiang) **2** *Cochylis discerta* Razowski, female (Sunan, Gansu) **3** *Cochylis dubitana* (Hübner), male (Tahe, Heilongjiang) **4** *Cochylis faustana* (Kennel), male (Beitun, Xinjiang) **5** *Cochylis posterana hyrcana* (Toll), male (Tacheng, Xinjiang) **6** *Cochylis triangula* sp. n., holotype, male (Daozhen, Guizhou). (scales = 2.5 mm).

Adult (Fig. 1) with wingspan 10.5−13.0 mm. This species is very similar to *Cochylis hybridella* (Hübner, [1813]), but *Cochylis atricapitana* can be distinguished by the pocket formed by the seventh sternum sclerotized on dorsal surface, the antrum composed of two connected rectangular plates and not bearing cylinder-shaped structure in the female genitalia (Fig. 12). In *Cochylis hybridella*, the pocket formed by the seventh sternum is membranous, the antrum is semicircular and bears a nearly cylinder-shaped structure in the female genitalia.

#### Distribution.

China (Xinjiang), Europe.

### Cochylis discerta

Razowski, 1970

http://species-id.net/wiki/Cochylis_discerta

Figs 2 , 7 , 13

Cochylis discerta Razowski, 1970: 431. TL: Mongolia (Cantral [Tov] Province, 11 km S Zosijn Davaa Pass).

#### Material examined.

**CHINA: Shanxi Province:** 1 ♀, Xiyao Village, Ningwu County (39°00'N, 112°18'E), 1475 m, 21.vii.2011, leg. Shulian Hao and Jiayu Liu. **Inner Mongolia:** Mt. Helan, Azuoqi (38°48'N, 105°52'E), 2200 m, 10.viii.2011, leg. Lixia Li and Yinghui Mou. **Gansu Province:** 2 ♂♂, 2 ♀♀, Sunan Autonomous County (38°50'N, 99°36'E), 2251 m, 16.viii.2007, leg. Feng Yang and Hanguang Gao.

#### Description.

Adult (Fig. 2) with wingspan 10.0−14.5 mm.

**Female genitalia** (Fig. 13). Papilla analis gradually narrowed posteriorly, about 1/3 length of apophysis posterioris. Apophysis anterioris about 3/5 length of apophysis posterioris. Sterigma a heavily sclerotized ring. Antrum heavily sclerotized, about 1/4 length of corpus bursae, anterior 1/3 curved in hook shape; ductus bursae extremely short, membranous. Corpus bursae elongate oval, membranous, densely suffused with tiny spines; ductus seminalis arising from posterior part of corpus bursae.

**Figures 7–10.**
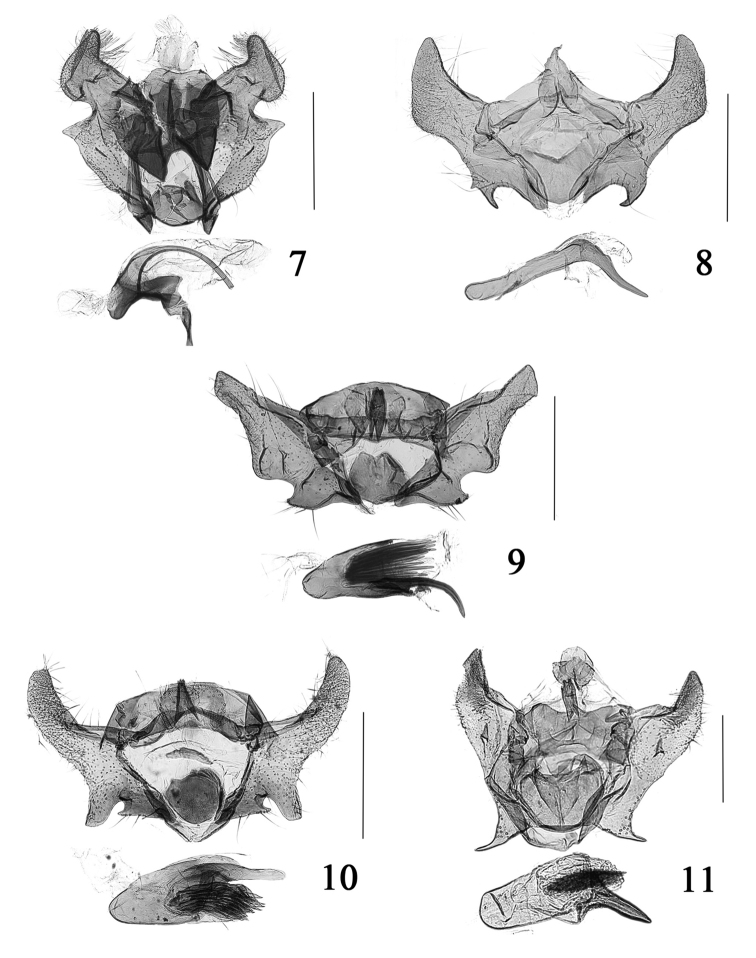
Male genitalia of *Cochylis* spp. **7** *Cochylis discerta* Razowski, slide No. SYH11644 **8** *Cochylis dubitana* (Hübner), slide No. SYH10244 **9** *Cochylis faustana* (Kennel), slide No. SYH11447 **10** *Cochylis posterana hyrcana* (Toll), slide No. SYH11434 **11** *Cochylis triangula* sp. n., paratype, slide No. SYH10200. (scales: 0.5 mm).

**Figures 12–16.**
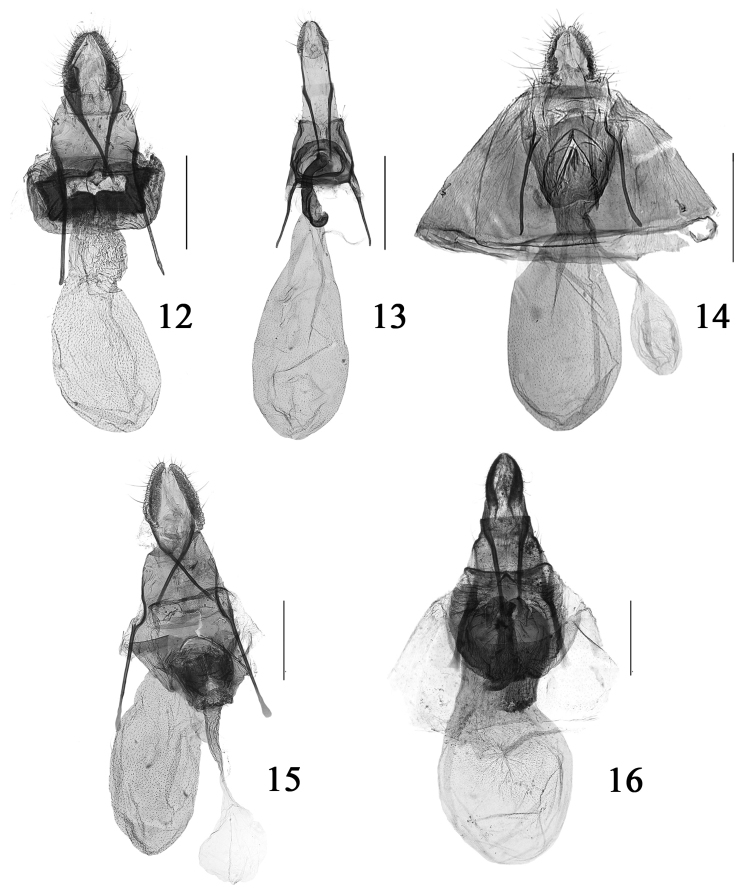
Female genitalia of *Cochylis* spp. **12** *Cochylis atricapitana* (Stephens), slide No. SYH11456 **13** *Cochylis discerta* Razowski, slide No. SYH11573 **14** *Cochylis faustana* (Kennel), slide No. SYH10228 **15** *Cochylis posterana hyrcana* (Toll), slide No. SYH11445 **16** *Cochylis triangula* sp. n., paratype, slide No. SYH11700. (scales: 0.5 mm).

#### Diagnosis.

This species can be distinguished from its congeners by the broad transtilla and the absence of a median process in the male genitalia (Fig. 7), and the ring-shaped sterigma and heavily sclerotized antrum in the female genitalia. *Cochylis discerta* Razowski, 1970 is very similar to *Cochylis psychrasema* (Meyrick, 1937), but *Cochylis discerta* can be distinguished by the ventral margin of the valva without a distal process in the male genitalia and the ductus bursae about 1/3 length of the corpus bursae in the female genitalia. In *Cochylis psychrasema*, the ventral margin of the valva bears a long spine-shaped process in the male genitalia and the ductus bursae is about 1/7 length of the corpus bursae in the female genitalia.

#### Distribution.

China (Gansu, Inner Mongolia, Shanxi), Mongolia.

#### Note.

The female is described here for the first time.

### Cochylis dubitana

(Hübner, [1799])

http://species-id.net/wiki/Cochylis_dubitana

Figs 3 , 8

Tortrix dubitana Hübner, [1799]: pl. 12, fig. 71. TL: Europe.
Tortrix ambiguana Frölich, 1828: 53. TL: Germany (Würtemberg).
Lobesia baseirufana Bruand, 1850: 99. TL: France.
Simaethis albidana Walker, 1866: 1807.
Cochylis dubitana (Hübner, [1799]): Razowski, 1960: 316.
Cochylis islandicana Björnsson, 1968: 24. TL: Iceland (Öraefum and Hreoarvatn).

#### Material examined.

**CHINA: Heilongjiang Province:** 2 ♂♂, Yongan Village, Tahe County (45°21N, 131°25'E), 28−29.vii.2009, leg. Weichun Li and Jiayu Liu.

#### Diagnosis.

Adult (Fig. 3) with wingspan 15.5 mm. This species is similar to *Cochylis hybridella*, but *Cochylis dubitana* can be distinguished by the sacculus not extended basally and the juxta slightly concave on the posterior margin in the male genitalia (Fig. 8). In *Cochylis hybridella*, the sacculus is extended to a hook-shaped process at the base and the posterior margin of the juxta is protruded posterolaterally in the male genitalia.

#### Distribution.

China (Heilongjiang), Europe.

### Cochylis faustana

(Kennel, 1919)

http://species-id.net/wiki/Cochylis_faustana

Figs 4 , 9 , 14

Phalonia faustana Kennel, 1919: 73. TL: Dscharkent [Russia] (Illi-Gebiet).
Cochylis faustana (Kennel, 1919): Razowski, 1968: 143.

#### Material examined.

**CHINA: Inner Mongolia:** 2 ♀♀, Erdaoqiao, Ejinaqi (41°58'N, 101°04'E), 927 m, 17−18.vii.2006, leg. Xinpu Wang and Xiangfeng Shi. **Xinjiang Uyghur Autonomous Region:** Beitun (47°18'N, 87°48'E): 5 ♂♂, 1 ♀, 530 m, 20.vii.1994, leg. Houhun Li and Hongyan Qin, 2 ♂♂, 512 m, 20.vii.2007, leg. Xinpu Wang; 1 ♂, Jinghe County (44°39'N, 82°56'E), 22.viii.1994, leg. Duoliken.

#### Diagnosis.

Adult (Fig. 4) with wingspan 8.0−9.5 mm. This species is similar to *Cochylis hybridella*, but *Cochylis faustana* can be distinguished by the short stripe-shaped median process of the transtilla about 1/3 length of the transtilla, and the phallus with more than ten cornuti in the male genitalia (Fig. 9); the seventh sternum not forming a membranous pocket, the antrum almost as long as wide, without cylinder-shaped structure at middle in the female genitalia (Fig. 14). In *Cochylis hybridella*, the median process of the transtilla is somewhat broad on basal 3/4 and slender on the distal 1/4, about 1/2 the length of the transtilla, and the slender phallus curves orthogonally and does not have cornutus in the male genitalia; the seventh sternum forms a membranous pocket, the length of the antrum is about 2/3 of width and bears a nearly cylinder-shaped structure at middle in the female genitalia.

#### Distribution.

China (Inner Mongolia, Xinjiang), Russia.

### Cochylis posterana hyrcana

(Toll, 1948)

http://species-id.net/wiki/Cochylis_posterana_hyrcana

Figs 5 , 10 , 15

Phalonia posterana hyrcana Toll, 1948: 112. TL: Iran (Kuh i Mirabi-Gebirge).
Cochylis posterana hyrcana (Toll, 1948): Razowski, 1970: 419.

#### Material examined.

**CHINA:** **Gansu Province:** 1 ♀, Mt. Xinglong, Yuzhong County (35°53'N, 104°06'E), 2178 m, 21.viii.2007, leg. Feng Yang and Hanguang Gao. **Xinjiang Uyghur Autonomous Region:** 20 ♂♂, 9 ♀♀, Abudula Village, Tacheng County (46°46'N, 82°59'E), 30.vii−23.viii.1990, leg. Jinfu Li; 1 ♀, Kuerdening, Gongliu County (43°28'N, 82°13'E), 1480 m, 4.viii.2007, leg. Xinpu Wang; 5 ♂♂, 2 ♀♀, Shirengou, Miquan City (43°58'N, 87°41'E), 1121 m, 11.viii.2007, leg. Xinpu Wang; 2 ♀♀, Nalati, Xinyuan County (43°21'N, 84°01'E), 1562 m, 6.viii.2007, leg. Xinpu Wang.

#### Diagnosis.

Adult (Fig. 5) with wingspan 12.0−15.0 mm. This species is similar to *Cochylis dubitana*, but *Cochylis posterana hyrcana* can be distinguished by the median process of the transtilla nearly triangular, the sacculus with a small terminal process, and the phallus with more than ten cornuti in the male genitalia (Fig. 10). In *Cochylis dubitana*, the median process of the transtilla is nearly triangular on basal 3/4 and slender on distal 1/4, the sacculus does not bear terminal process, and the phallus does not have cornutus in the male genitalia. The female genitalia (Fig. 15) are similar to those of *Cochylis hybridella*, but *Cochylis posterana hyrcana* can be distinguished by the antrum consisting of two rounded plates close to each other and the absence of a cylinder-shaped structure at middle. In *Cochylis hybridella*, the antrum is a nearly rounded plate bearing an approximately cylinder-shaped structure at middle in the female genitalia.

#### Distribution.

China (Gansu, Xinjiang), Iran. The nominate subspecies occurs in Europe.

#### Remarks.

The type locality of *Cochylis posterana posterana* Zeller, 1847 is Hungary. Then it was reported to occur in different countries of Europe. *Cochylis posterana hyrcana* can be distinguished from *Cochylis posterana posterana* by the forewing with median fascia conspicuous anteriorly, the median process of the transtilla nearly triangular in the male genitalia. In *Cochylis posterana posterana*, the median fascia is invisible anteriorly in the forewing (Razowski 1970: Taf. 26, Fig. 273), and the median process of the transtilla is uniform in width in the male genitalia (Razowski 1970: Taf. 106, Fig. 273).

### Cochylis triangula sp. n.

urn:lsid:zoobank.org:act:C1B99980-0941-427F-8978-C26E6DB6FD82

http://species-id.net/wiki/Cochylis_triangula

Figs 6 , 11 , 16

#### Type material.

**CHINA: Holotype** ♂**, Guizhou Province:** Guocun Village, Daozhen County (28°53'N, 107°36'E), 1300 m, 21.viii.2004, leg. Yunli Xiao, genitalia slide No. SYH10220.

**Paratypes:** 1 ♀, same data as for holotype. **Yunnan Province:** 2 ♂♂, Xiaoheishan, Longling County (24°35'N, 98°41'E), 2300 m, 10.viii.2005, leg. Yingdang Ren.

#### Description.

Adult (Fig. 6) with wingspan 15.5−17.0 mm. Vertex and frons pale yellowish white. Antenna yellowish brown, mixed with brownish black scales. Labial palpus slender, about 1.5 times length of eye’s diameter, yellowish brown on outer surface, yellowish white on inner surface. Thorax and tegula pale yellowish white, tegula with a brownish black spot at base. Forewing with costal margin straight, apex protruded, termen oblique. Ground color pale yellowish white; costal margin mixed with small brownish black spots on basal half, with brownish black spots at base and at basal 1/4, with a short and thin stripe at distal 1/6; basal patch occupying basal 1/4 of forewing, consisting of thin grayish black stripes; median fascia from middle of costal margin extending obliquely to middle of dorsum, grayish black with sparse ochreous yellow, anterior 1/4 oblique outward, somewhat narrow, anterior 1/4 to 1/2 disappeared, posterior half somewhat broad, oblique inward; subapical fascia a brownish black stripe along termen, mixed with ochreous yellow scales; tornus with a large brownish black patch; dorsum with small brownish black spots; cilia pale brown. Hindwing and cilia grayish white. Fore- and midlegs brownish black, with yellowish white rings; hindleg yellowish white. Abdomen grayish brown.

#### Male genitalia

(Fig. 11). Socius about 2/3 length of median process of transtilla. Valva short and broad, outer margin slightly convex, dorsal corner slightly pointed, with a spine-shaped process at basal 1/3 near outer margin; costa concave; transtilla broad, gradually narrowed from base to middle, median process about 1/3 length of transtilla. Sacculus heavily sclerotized, almost same length as costa, dorsal margin straight, ventral margin protruded subtriangularly, apex pointed and hook-shaped; vinculum slender, connected with membrane ventrally. Juxta nearly semicircular, anterior margin rounded, posterior margin straight. Phallus about two times length of costa, basal 3/5 stout, distal 2/5 thick thorn-shaped; cornutus a cluster short thin spines, about 2/5 length of phallus.

#### Female genitalia

(Fig. 16). Papilla analis somewhat small, about 1/2 length of apophysis posterioris. Apophysis anterioris slightly shorter than apophysis posterioris. Sterigma weakly sclerotized, weakly defined. Seventh sternum forming a special membranous pocket, surrounding antrum. Antrum heavily sclerotized, nearly rectangular, with a heavily sclerotized vertical band at middle. Ductus bursae short and broad, about 1/2 length of antrum, weakly sclerotized, with vertical wrinkles. Corpus bursae oval.

#### Diagnosis.

This species is similar to *Cochylis posterana hyrcana*, but *Cochylis triangula* sp. n. can be distinguished by the sacculus with ventral margin protruded triangularly, and the cornutus being a cluster of short and thin spines in the male genitalia; the nearly rectangular antrum with a heavily sclerotized vertical band at middle in the female genitalia. In *Cochylis posterana hyrcana*, the ventral margin of the sacculus is straight, and the cornutus consists of a bundle of more than ten thin spines in the male genitalia; the antrum is composed of two rounded plates close to each other and the absence of the sclerotized vertical band at middle in the female genitalia.

#### Distribution.

China (Guizhou, Yunnan).

#### Etymology.

The specific name is the feminine form of the Latin adjective *triangulus*, meaning triangular, referring to the triangular sacculus.

## Supplementary Material

XML Treatment for Cochylis

XML Treatment for Cochylis atricapitana

XML Treatment for Cochylis discerta

XML Treatment for Cochylis dubitana

XML Treatment for Cochylis faustana

XML Treatment for Cochylis posterana hyrcana

XML Treatment for Cochylis triangula
